# Healthcare Workforce Well-Being Within the Health Promoting Hospitals and Health Services Framework: A Narrative Review and Organizational Case Example

**DOI:** 10.3390/healthcare14203517

**Published:** 2026-10-19

**Authors:** Fabio D’Antoni, Laura Brunelli, Enrico Scarpis, Cristina Aguzzoli, Alessio Matiz, Cristiano Crescentini

**Affiliations:** 1 Child and Family Service, Friuli Centrale Healthcare University Trust, 33100 Udine, Italy; 2 Department of Medicine, University of Udine, 33100 Udine, Italy; laura.brunelli@asufc.sanita.fvg.it; 3 Accreditation, Quality, and Clinical Risk Unit, Friuli Centrale Healthcare University Trust, 33100 Udine, Italy; enrico.scarpis@asufc.sanita.fvg.it; 4 Hospital of Latisana-Palmanova, Friuli Centrale Healthcare University Trust, 33057 Palmanova, Italy; 5 HPH Network of Friuli Venezia Giulia, Regional Authority for Coordination of Health Trust, 33100 Udine, Italy; cristina.aguzzoli@arcs.sanita.fvg.it; 6 Department of Human Sciences, Link Campus University, 00165 Rome, Italy; a.matiz@unilink.it; 7 Department of Languages and Literatures, Communication, Education and Society, University of Udine, 33100 Udine, Italy; cristiano.crescentini@uniud.it

**Keywords:** Health Promoting Hospitals, healthcare workforce, workplace health promotion, employee well-being, organizational health promotion, implementation, salutogenesis

## Abstract

**Background/Objectives**: Healthcare workforce health and well-being are explicitly included within the Health Promoting Hospitals and Health Services (HPH&HS) framework, but the literature has not been synthesized through a dedicated workforce-centered lens. This narrative review examined how workforce health is conceptualized within HPH&HS, operationalized through standards and organizational processes, addressed through workforce-oriented interventions and outcomes, and shaped by organizational conditions influencing implementation. **Methods**: PubMed/MEDLINE, Scopus, Web of Science Core Collection, and CINAHL Complete were searched for publications from 1986 to 2026. Of 323 records identified, 148 were screened after deduplication, 60 full texts were assessed, and 51 reports were included. Evidence was synthesized across four domains: conceptual foundations, standards and governance, workforce interventions and outcomes, and implementation conditions. **Results**: Workforce health emerged as an intrinsic HPH&HS dimension, with healthcare professionals positioned as both targets and agents of health promotion. Workforce health was increasingly formalized through HPH&HS standards, governance structures, and organizational processes. Leadership, resources, workforce participation, competencies, integration into routine practice, and supportive policy environments emerged as recurrent implementation conditions. Evidence addressed health behaviors, occupational health and safety, psychological well-being, and job satisfaction, as well as organizational culture and staff participation. However, the empirical evidence was predominantly cross-sectional and heterogeneous, with limited controlled or longitudinal research linking HPH&HS implementation to improved workforce health. **Conclusions**: HPH&HS provides a conceptual and organizational framework for workforce health promotion, but implementation evidence is stronger than evidence for direct and sustained health improvements. Future research should distinguish implementation outcomes, organizational or work-environment indicators, and workforce health outcomes using longitudinal and controlled designs.

## 1. Introduction

The Health Promoting Hospitals and Health Services (HPH&HS) framework represents an organizational approach to health promotion rooted in the settings approach [1,2]. The settings approach conceptualizes the hospital not merely as a passive venue for the delivery of specific health services, but as a complex organizational setting in which environmental, organizational, and individual factors interact [3].

Originally conceptualized by the World Health Organization (WHO) in the early 1990s, the HPH&HS framework was designed to implement the 1986 Ottawa Charter’s mandate of “reorienting health services” [1,4]. This shift requires healthcare systems to move beyond their traditional curative and clinical responsibilities toward a more holistic mandate that addresses the broader social, economic, and environmental determinants of health [1,5].

In its current definition, HPH&HS is characterized by the systematic reorientation of governance models, structures, processes, and culture to optimize health gains for patients, staff, and the populations served, while supporting sustainable societies. While the movement initially focused on acute hospital settings, it transitioned in 2007 to include the broader spectrum of health services, aligning with primary healthcare principles and recognizing the necessity of linking hospitals with community-wide health initiatives [1]. The HPH&HS literature encompasses organizational processes, patient-related outcomes, health-promotion activities, and workforce-related dimensions [2,4,5].

Salutogenesis provides an important conceptual perspective for interpreting the HPH&HS approach. Originally conceptualized by Aaron Antonovsky, this theoretical framework shifts the focus from the origins of disease (pathogenesis) to the origins of health. Rather than viewing health as a binary state of being ‘sick’ or ‘healthy,’ the salutogenic paradigm conceptualizes it as a dynamic position along a health ease/dis-ease continuum [6,7]. Within this perspective, HPH standards can be understood as organizational tools intended to support health-promoting conditions for patients, communities, and the workforce. More broadly, the HPH&HS framework is consistent with a salutogenic perspective in emphasizing healthcare organizations as settings in which environmental, organizational, and individual resources for health can be strengthened [8]. From this perspective, the reorientation of organizational governance, structures, processes, and culture extends the scope of health promotion beyond the delivery of clinical care to include the conditions within which health is produced and maintained [8].

Previous reviews have examined the development of the HPH movement, the organizational changes required for its implementation, and the factors facilitating or hindering the establishment of health-promoting hospitals [9,10,11]. Yaghoubi et al. [11], for example, systematically synthesized factors affecting HPH implementation and development within an organizational quality-management framework, identifying leadership, resources, staff training and participation, and a healthy work environment among relevant implementation dimensions. However, workforce health was embedded within a broader analysis of HPH implementation rather than examined as the primary focus of the review.

Despite these contributions, healthcare workforce health and well-being have not been synthesized through a dedicated workforce-centered lens across the conceptual, organizational, implementation, and outcome-oriented HPH&HS literature. Evidence relevant to the workforce is distributed across conceptual contributions, standards and organizational assessments, implementation studies, and investigations of staff health behaviors, working conditions, and workforce-oriented interventions. Bringing these strands together may clarify not only how workforce health has been represented within the HPH&HS framework, but also the extent to which its formal inclusion in HPH&HS principles and standards has translated into organizational practice and measurable workforce outcomes.

Accordingly, the present narrative review aims to examine healthcare workforce health and well-being through the specific lens of the HPH&HS framework. It seeks to (1) examine how workforce health has been conceptualized within the theoretical development of HPH&HS; (2) describe how it has been operationalized through HPH&HS standards, governance, and organizational processes; (3) synthesize the available evidence on workforce-focused interventions and health- or work-related outcomes; and (4) identify organizational conditions that facilitate or constrain implementation. To complement the literature synthesis, the experience of the Friuli Centrale Healthcare University Trust (ASUFC), Italy, is presented separately as an organizational case example illustrating how different workforce-oriented initiatives can be situated within a common HPH&HS governance framework.

## 2. Materials and Methods

### 2.1. Review Design

This study was designed as a narrative review with a structured literature search, aimed at examining how healthcare workforce health and well-being have been conceptualized, investigated, and implemented within the Health Promoting Hospitals and Health Services (HPH&HS) framework. Rather than addressing workforce well-being as an isolated individual outcome, the review focused on its position within the broader organizational and salutogenic orientation of HPH&HS.

The literature synthesis was subsequently used as the conceptual framework for interpreting an organizational case example developed within a large Italian public healthcare organization. The review component and the organizational case example were therefore treated as distinct but complementary parts of the study.

### 2.2. Literature Search Strategy

A structured literature search was conducted on 18 August 2026 across four electronic databases: PubMed/MEDLINE, Scopus, Web of Science Core Collection, and CINAHL Complete. The search covered publications from 1986, corresponding to the year of the Ottawa Charter for Health Promotion, to 2026.

The search strategy combined terms referring to the HPH&HS framework with terms related to healthcare workers, workplace health, and workforce well-being. The following core search string was adapted, where necessary, to the syntax of each database: (“health promoting hospital*” OR “health-promoting hospital*” OR “health promoting health service*” OR “health-promoting health service*”) AND (“healthcare worker*” OR “health care worker*” OR “healthcare professional*” OR “health personnel” OR staff OR employee* OR workforce OR workplace OR “occupational health” OR wellbeing OR “well-being” OR burnout OR “job satisfaction” OR “work engagement” OR stress OR resilience).

No restrictions based on study design were applied at the search stage in order to capture the empirical, implementation-oriented, conceptual, and review literature relevant to the development and application of the HPH&HS approach.

The searches identified 323 records: 87 from PubMed/MEDLINE, 127 from Scopus, 68 from Web of Science Core Collection, and 41 from CINAHL Complete.

### 2.3. Eligibility Criteria

Publications were considered potentially eligible when they explicitly addressed the HPH/HPH&HS framework, or an intervention or organizational approach clearly attributable to it, and included a substantive component concerning healthcare workforce health, well-being, or working conditions. Relevant domains included occupational and workplace health, psychological well-being and distress, stress and burnout, job satisfaction and work engagement, resilience, staff health behaviors, workplace health promotion, organizational resources and working conditions, and interventions designed to promote staff health and well-being.

Empirical studies, implementation and evaluation studies, reviews, and conceptual or theoretical contributions were considered eligible when they provided information relevant to the review question. Studies focusing exclusively on patients, families, or community populations without a substantive workforce component were excluded. Studies addressing healthcare worker stress, burnout, resilience, or well-being outside an identifiable HPH/HPH&HS framework were also excluded. Particular attention was paid to studies in which the term health-promoting hospital referred to a local designation for a healthcare facility rather than to the WHO HPH/HPH&HS framework; these were considered outside the scope of the review.

### 2.4. Record Management and Screening

All retrieved records were merged into a single database. Deduplication was conducted sequentially using normalized Digital Object Identifiers (DOIs), PubMed identifiers (PMIDs), and normalized titles, followed by manual inspection of potentially matching records based on title, authorship, publication year, and translated or alternative title variants. This procedure identified 175 duplicate records, leaving 148 unique records for title and abstract screening.

Title and abstract screening and full-text eligibility assessment were conducted by the first author. Records classified as uncertain, as well as borderline cases identified during screening, were subsequently re-examined to ensure consistent application of the predefined eligibility criteria. Full-text eligibility assessment was performed using the same criteria. Records were classified as include, exclude, or uncertain. A conservative approach was adopted: when the available bibliographic information or abstract did not permit a sufficiently confident assessment of either the relationship with the HPH/HPH&HS framework or the relevance to healthcare workforce well-being, the record was retained as uncertain for subsequent full-text assessment.

Following title and abstract screening, 82 records were excluded and 66 records were retained for full-text retrieval and eligibility assessment.

### 2.5. Full-Text Assessment and Data Extraction

Full texts were sought for all 66 records retained after title and abstract screening. Six reports could not be retrieved despite reasonable attempts through institutional library resources, publisher websites, and available repositories and were therefore classified as not retrieved. The remaining 60 reports underwent full-text eligibility assessment.

Eligibility at the full-text stage was determined according to the same two core criteria applied during screening: (1) the publication had to be substantively embedded within the Health Promoting Hospitals and Health Services (HPH/HPH&HS) framework, rather than merely referring to health promotion in healthcare settings; and (2) it had to include a meaningful component related to healthcare workforce health, well-being, working conditions, or organizational processes directly relevant to their promotion. Publication type and language were not used as automatic exclusion criteria. Conceptual papers, reviews, editorials, conference contributions, and publications in languages other than English were retained when sufficient substantive information relevant to the review question could be reliably assessed.

Nine reports were excluded following full-text assessment. Exclusion resulted from the absence of a sufficiently substantive HPH/HPH&HS framework, the absence of a meaningful workforce-related component, or insufficient substantive content to contribute to the review question. Consequently, 51 reports were retained for the narrative synthesis.

For all included publications, data were extracted into a standardized matrix developed specifically for the review. Extracted information included bibliographic characteristics, country and healthcare setting, publication or study design, population and sample characteristics, the HPH framework or standards applied, the nature of the workforce component, interventions or organizational processes, workforce-related outcomes or constructs, main findings, reported barriers and facilitators, organizational implications, and the principal contribution of each publication to the review question. Locations within the source documents were also recorded where appropriate to facilitate verification during synthesis.

**Figure 1:**
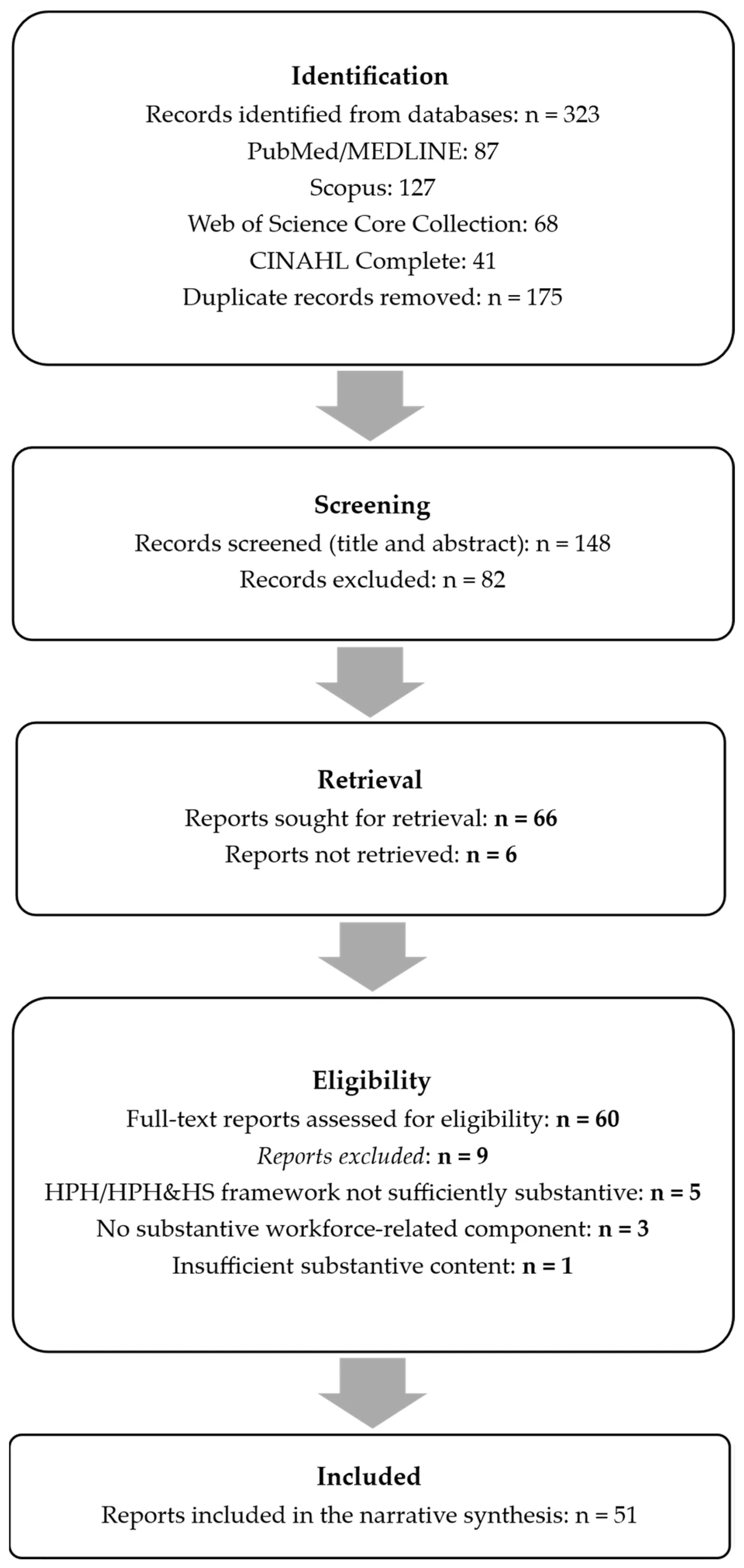
Flow diagram of the literature search and selection process. Records were identified through four electronic databases, deduplicated, screened by title and abstract, and subsequently assessed for full-text eligibility. HPH, Health Promoting Hospitals; HPH&HS, Health Promoting Hospitals and Health Services.

Potentially overlapping publications were identified during data extraction. When multiple reports originated from the same organizational initiative, hospital network, or study population but provided complementary information, they were retained as separate reports but treated as related evidence rather than as independent organizational experiences during interpretation. The study selection process is summarized in Figure 1.

### 2.6. Narrative Synthesis

A narrative synthesis was undertaken because of substantial heterogeneity across the included literature in study design, healthcare setting, workforce population, HPH implementation strategy, and reported outcomes. Statistical pooling was therefore neither planned nor considered appropriate.

The synthesis followed a structured and iterative process based on the standardized extraction matrix. An initial conceptually informed framework was used to organize the literature around four broad domains: (1) conceptual foundations and organizational change; (2) HPH standards, governance, and quality assessment; (3) workforce health, well-being, interventions, and outcomes; and (4) organizational conditions influencing implementation. These domains were used as sensitizing categories rather than fixed analytical themes and were refined during cross-study comparison according to patterns emerging from the included literature.

Particular attention was given to distinguishing organizational and implementation outcomes—including HPH standards compliance, organizational capacity, policy integration, implementation readiness, staff participation, and quality-management processes—from direct workforce outcomes, such as health behaviors, occupational health and safety, psychological health and stress-related outcomes, quality of life, job satisfaction, and other indicators of staff well-being. This distinction was maintained throughout the synthesis to avoid interpreting successful implementation of HPH structures or standards as evidence, in itself, of improved workforce health.

Reported barriers and facilitators were mapped across the included publications to identify recurrent organizational conditions associated with HPH implementation. These mappings were used descriptively to support the narrative synthesis and were not interpreted as quantitative estimates of prevalence or effect. Similarly, greater interpretive weight was given to convergence across independent settings and study designs and, where available, to directly measured workforce outcomes rather than solely to conceptual propositions or perceived benefits.

Where several publications originated from the same organizational project, hospital network, or overlapping study population, their findings were considered jointly in the synthesis to minimize double counting and overrepresentation of individual implementation experiences.

## 3. Results

### 3.1. Characteristics of the Included Literature

A total of 51 reports published between 1999 and 2025 were included in the narrative synthesis [12,13]. The literature showed substantial geographical, methodological, and conceptual heterogeneity. Most reports originated from Asian healthcare systems, with Iran (*n* = 15; e.g., [14,15,16,17,18,19,20,21,22,23,24,25,26,27,28]) and Taiwan (*n* = 11; e.g., [29,30,31,32,33,34,35,36,37,38,39]) accounting for approximately half of the included literature. Three reports originated from South Africa [40,41,42], two from Pakistan [43,44], and individual reports were identified from Australia [3], Belgium [45], Canada [12], China [46], the Czech Republic [13], Estonia [47], Ghana [48], Greece [49], Indonesia [50], Japan [51], and Lithuania [52], as well as a study involving healthcare organizations in Germany and Austria [53]. The corpus also included multicountry studies [54,55] and international or European conceptual contributions [2,9,56,57]. The main characteristics of the included literature are summarized in Table 1. Detailed characteristics of each of the 51 included reports, including healthcare setting, study design, population, HPH framework, workforce focus, and main workforce-relevant contribution, are provided in Table A1.

The literature addressing health promotion in hospital settings expanded markedly over time. Only two reports in the selected corpus were published before 2000 [12,58] and eight during 2000–2009 [2,3,9,46,47,49,53,56], whereas 29 were published between 2010 and 2019 (e.g., [10,11,15,18,19,20,21,26,27,30,31,32,34,35,36,37,38,39,40,41,42,43,44,45,52,54,55]) and 12 between 2020 and 2025 [13,14,16,17,23,24,25,28,29,48,50,51]. This temporal distribution reflects the progressive development of the HPH framework, transitioning from early conceptual and organizational formulations (e.g., [2,3,9,12]) towards a broader empirical literature addressing implementation, organizational capacity, standards, and workforce-related outcomes (e.g., [21,29,34,38,54,55]).

Study designs were heterogeneous and predominantly observational. Cross-sectional surveys and organizational self-assessments represented the largest methodological group (*n* = 26; [13,15,16,17,18,20,22,23,24,25,26,27,29,30,31,32,33,34,35,36,38,39,42,47,51,52]), followed by qualitative or predominantly qualitative mixed-method studies (*n* = 10; e.g., [14,19,21,37,43,44,45,48,54]), conceptual or narrative review contributions (*n* = 9; e.g., [2,3,9,12,56,57,58]). The remaining literature comprised organizational case or implementation studies (e.g., [40,41,53], one systematic review [11], and one randomized implementation trial [55]). Consequently, the evidence base was primarily suited to examining organizational processes, implementation conditions, workforce needs, and associations rather than establishing causal effects of HPH implementation on employee health.

The role assigned to the healthcare workforce also varied considerably across reports. In some studies, workforce health constituted the primary object of investigation, with analyzed outcomes including health behaviors and tobacco use [13,31,41,42], occupational health and safety [14,30,39], psychological health and stress-related indicators [24,28,30], job satisfaction and organizational culture [17,28], and active participation in workplace health promotion activities [29,31,33]. In others, workforce-related issues were examined as core components of broader HPH implementation, particularly through the WHO healthy-workplace standard [15,16,18,20,25,27,52], organizational capacity [34,35,36], staff participation and empowerment [12,45], professional training [19,48], or organizational policies and governance [53]. Based on their principal contribution to the present review, 17 reports were primarily classified as addressing implementation conditions, 14 as standards and governance, 14 as workforce interventions or outcomes, and six as conceptual foundations. The classification of individual reports across these domains is provided in Table A1.

The 51 included publications should not, however, be interpreted as representing 51 independent organizational experiences. Several reports originated from the same HPH initiatives, hospital networks, or partially overlapping populations and provided complementary perspectives on the same implementation contexts. For example, multiple publications described different components of the South African HPH pilot initiative [40,41,42], while several Taiwanese studies drew on national HPH networks or related hospital populations. This latter group includes a cluster of studies utilizing a single 2011 national survey of 100 hospitals to address distinct workforce sub-topics [29,30,31,39] and a series of reports evaluating the same cohort of 52 Taiwanese HPH coordinators [33,34,35,36]. Such reports were retained because they contributed distinct thematic information, but they were considered jointly when interpreting the overall evidence to avoid overrepresenting individual organizational experiences.

### 3.2. Workforce Health as an Intrinsic Dimension of the HPH&HS Model

The six reports primarily classified as conceptual foundations consistently positioned workforce health as an intrinsic component of the HPH&HS model rather than as an ancillary program directed at employees. Foundational contributions consistently extended the scope of hospital health promotion beyond patient-oriented interventions, conceptualizing healthcare organizations as settings in which the health of patients, staff, the organization itself, and the surrounding community are interconnected [2,9,12,56,57,58]. Within this perspective, the healthcare workforce occupies a dual position: staff members are both agents responsible for delivering health-promoting practices and a population whose own health and working conditions should be actively protected and promoted.

This conceptualization directly reflects the settings-based orientation underlying the HPH movement [2,9,12]. Rather than locating responsibility for workforce health exclusively at the individual level, the literature progressively situated it within the broader organizational environment [9,12,57]. Working conditions, organizational culture, leadership, participation in decision-making, professional development, and the physical and psychosocial characteristics of the workplace were therefore treated as relevant components of health promotion interconnected [9,12,57,58]. Early organizational perspectives accordingly emphasized the need to move beyond isolated health-promotion projects towards the integration of health promotion into hospital structures, routines, professional roles, and organizational development processes [2,9,12,57].

The literature also showed a progressive shift from an individual risk-factor orientation towards a more comprehensive understanding of workforce health [9,12]. In this broader perspective, staff-directed health promotion encompasses not only lifestyle behaviors or the prevention of occupational risks, but also the organizational resources that enable healthcare workers to maintain health and functioning within demanding work environments [9,12,57]. Salutogenic approaches further reinforced this orientation by emphasizing resources, coping capacity, workability, and sense of coherence rather than focusing exclusively on disease prevention or risk reduction [57]. Workforce health promotion can therefore be understood as operating simultaneously at individual, interpersonal, organizational, and environmental levels [2,12,57].

A further recurrent feature was the close relationship between workforce health and organizational capacity for health promotion [9,12,57]. Staff knowledge and competencies, participation and empowerment, supportive leadership, organizational policies, and the availability of structures and resources for health promotion were repeatedly represented as conditions through which HPH principles could become embedded in everyday practice [2,9,12,56,57,58]. Consequently, the workforce dimension cannot be separated entirely from organizational development: the capacity of healthcare professionals to act as health promoters is partly dependent on whether the organization itself provides a supportive and health-promoting working environment [2,9,12,57].

Taken together, the included literature conceptualizes workforce health as a constitutive dimension of the HPH&HS model, rather than as an auxiliary or secondary institutional concern [2,9,12]. The healthcare workforce is not merely an instrument through which health promotion is delivered to patients, but represents one of the three primary target populations whose health, safety, and general welfare the organization is explicitly expected to protect and promote [2,9,12,57,58]. This conceptual foundation provides a robust theoretical basis for the subsequent operationalization of workforce-directed health promotion within HPH standards and organizational quality assessment frameworks, examined in the following section [2,9,57].

### 3.3. From HPH&HS Standards to Organizational Institutionalization

The 14 reports classified within the standards and governance domain examined how HPH&HS principles were translated into measurable standards, organizational assessment procedures, and management processes. Much of this literature was structured around the five WHO HPH standards—management policy, patient assessment, patient information and intervention, promoting a healthy workplace, and continuity and cooperation—thereby providing an operational framework through which health promotion could be assessed at the organizational level [15,16,17,18,20,23,24,25,26]. Within this framework, workforce health was addressed most explicitly through Standard 4, Promoting a Healthy Workplace, which included working conditions and occupational risks as well as staff awareness, training, participation in organizational processes, access to health-promotion initiatives, and assessment of staff needs. For example, the WHO-based instrument used by Afshari et al. (2016) included occupational and physical health records, staff participation in hospital committees, smoking-cessation programs, healthy-food policies, and workplace risk assessment within the healthy-workplace standard [15].

The standards-based assessments showed considerable heterogeneity in the extent to which these principles had been implemented. In the two hospitals examined by Hamidi et al. (2017), Promoting a Healthy Workplace showed limited implementation (31.24% on average), with substantial variation between the cardiovascular hospital (40.62%) and the women and parturition hospital (21.87%) [18]. In a broader assessment of nine educational hospitals, Afshari et al. (2016) reported an overall mean HPH score of 48.8 ± 9.8 (out of 100), with Standard 4 (Promoting a Healthy Workplace) scoring 56.2 ± 12.5, compared with 79.8 ± 13.5 for patient information and intervention [15]. Other assessments did not invariably identify Standard 4 as the weakest domain, indicating that workforce-related implementation deficits occurred within a broader pattern of uneven compliance across the HPH standards rather than representing a uniform hierarchy of weaknesses across settings [15,17,20,26].

Studies based on healthcare professionals’ perceptions provided further evidence of a gap between the existence of standards and their translation into everyday practice [20,52]. In Lithuania, Miseviciene and Zalnieraitiene [52] found generally limited staff awareness of several health-promotion activities and reported that only approximately one-third of respondents considered Standards 3 and 4 realistically integrable into daily practice. Awareness and perceived applicability also differed according to hospital size—with healthcare professionals in smaller facilities demonstrating significantly better knowledge and more positive views—as well as professional and managerial position. Similarly, Mahmoodi et al. [20] found that only 28% of participating health professionals considered the Promoting a Healthy Workplace standard applicable in their hospitals. Perceived applicability varied according to hospital characteristics (such as size and speciality) and previous knowledge of the HPH strategy, while managerial staff generally reported higher applicability scores than nurses or physicians. These findings suggest that formal standards alone do not ensure either workforce awareness or their perceived feasibility within routine organizational practice.

Other studies used the standards not only to measure compliance but also to identify priorities for organizational improvement. Sadeqi-Arani et al. [23], for example, combined assessment of the importance and performance of the five HPH standards across four hospitals. Performance differed significantly between hospitals, and Promoting a Healthy Workplace emerged as a critical area in three of the four organizations. The improvement strategies generated from the analysis included staff education in health promotion, diet and physical-activity programs for employees, and greater staff participation in health-promotion policies, evaluation, and organizational decision-making. Thus, the standards functioned not simply as descriptive measures but as instruments for identifying organizational gaps and directing subsequent action.

The relationship between HPH standards and organizational management was particularly explicit in Groene et al. (2009) [53]. Rather than treating HPH as a parallel program, the study combined HPH standards and strategies with the European Foundation for Quality Management (EFQM) model and the Balanced Scorecard to translate broad organizational goals into 20 strategic objectives with corresponding measures, responsibilities, targets, data-collection procedures, and action plans. This approach illustrates a deeper level of institutionalization in which health-promotion principles become connected to existing strategy, performance measurement, and quality-management systems [53].

The broader organizational requirements for such institutionalization were synthesized by Yaghoubi et al. (2019) [11]. Their systematic review identified a range of factors associated with HPH implementation and development, including leadership, policies and strategy, human resources, staff training and participation, organizational communication, partnerships, resource allocation, and organizational processes. The review emphasized the need for an established organizational structure capable of integrating health promotion with quality management, and contrasted approaches in which health promotion is simply added to existing hospital activities with approaches in which it is fully integrated into organizational structures and culture. The synthesis therefore extends the meaning of standards compliance from the achievement of individual indicators towards the development of comprehensive organizational capacity for health promotion [11].

More recent evidence suggests that the gap between formal inclusion and organizational integration remains relevant as HPH standards evolve and are adapted to different health systems. Al Aufa et al. [50], assessing the implementation of the Indonesian national HPH standards adapted from the WHO framework, reported that assessment (73.72%) and intervention (72.71%) obtained higher scores than policy and management (69.81%) and monitoring and evaluation (67.82%). Although the standards formally addressed patients, families, hospital staff, visitors, and the community, implementation in practice remained predominantly patient-focused, with comparatively limited attention to healthcare workers. The authors consequently emphasized management and policy, monitoring and evaluation, and the training of healthcare leaders and workers as priorities for strengthening HPH implementation.

Kawabata et al. (2025) broadened this perspective beyond hospitals by examining health-promotion activities across hospitals, clinics, and nursing-care facilities and organizing these activities in relation to the 2020 HPH Standards [51]. All participating facilities reported health-promotion activities, including activities directed towards staff, but their extent and organizational conditions varied according to facility type. This more recent application illustrates the extension of HPH&HS principles from hospital-specific assessment towards a broader organizational framework applicable across different healthcare and care settings [51].

Taken together, the 14 standards and governance reports describe a progression from formal specification to organizational institutionalization. Standards provide a means of defining and measuring the workforce dimension of HPH&HS, but the evidence indicates that their formal presence does not necessarily imply staff awareness, perceived applicability, participation, or consistent implementation. Deeper institutionalization was instead reflected in the incorporation of health promotion into organizational policy and strategy, management and quality systems, staff roles and competencies, resource allocation, monitoring and evaluation, and mechanisms for workforce participation. Within this process, Promoting a Healthy Workplace represents an explicit operational expression of workforce health within the HPH&HS framework, while its effective implementation depends on its integration into the wider organizational system.

### 3.4. Workforce-Focused Interventions and Outcomes

The 14 reports primarily classified within the workforce interventions and outcomes domain examined workforce health from complementary behavioral, occupational, psychosocial, and organizational perspectives [13,14,27,28,29,30,31,32,33,39,42,45,47,55]. The evidence base was methodologically heterogeneous and predominantly observational, including large cross-sectional workforce surveys [27,28,29,30,31,39,42], organizational assessments [33,45,47], repeated cross-sectional studies [13], implementation studies [14], and one randomized implementation trial [55]. Accordingly, the findings provide substantially stronger evidence regarding workforce needs, patterns, and associations than regarding the causal effects of HPH&HS implementation on employee health [32,55].

Health behaviors and participation in workplace health promotion constituted a prominent area of investigation across the empirical literature [29,31]. In a nationwide Taiwanese study involving 26,011 full-time nurses, Chen et al. [29] found that nurses employed in certified HPH hospitals were significantly more likely than those in non-HPH hospitals to report regular physical activity, recent general physical examinations, and cancer screening, as well as active participation in selected hospital-based health-promotion activities. However, these differences were not consistent across all health behaviors—with no significant variation observed in lecture attendance—and the cross-sectional design of the survey precluded any causal attribution to HPH certification [29]. Similarly, Chiou et al. [31], examining 59,396 hospital employees, identified substantial differences across occupational groups in both health behaviors and participation in hospital-run health-promotion activities. Nurses reported comparatively lower levels of physical activity, fruit and vegetable consumption, stress adaptation, and overall participation in health-promoting programs than other professional groups, while active participation itself was strongly associated with more favorable individual health behaviors.

Other studies used workforce assessment to identify specific health-promotion needs and establish baseline parameters prior to intervention [27,42]. Taghdisi et al. (2018) assessed health-promoting behaviors among employees and nurses at the beginning of HPH activities in an Iranian heart center, finding interpersonal relationships to be the strongest behavioral dimension (24.0 ± 3.0), whereas physical activity (15.0 ± 4.0) and stress management were comparatively weak [27]. In the South African HPH experience, Delobelle et al. (2012) similarly identified critical workforce-related lifestyle and environmental priorities, documenting widespread staff dissatisfaction with the range of available food choices (80%) and a strong consensus (72%) that the hospital needed to improve its supportive workplace conditions for healthy eating [42]. These studies primarily informed the systematic identification of staff needs and the development of localized intervention priorities, rather than demonstrating immediate clinical or health outcomes attributable to HPH implementation [27,42].

Tobacco use represented a further workforce-health outcome examined across several HPH contexts [13,29,42,47]. Králíková et al. (2025), using repeated anonymous cross-sectional surveys conducted in 2012 and 2023 in a large Czech HPH university hospital, reported a substantial decline in overall smoking prevalence among healthcare personnel from 38.4% to 14.5% [13]. Specifically, smoking rates fell from 15.86% to 6.75% among all physicians and from 44.59% to 17.64% among nurses. Nevertheless, the cross-sectional observational design, moderate response rates (25% in 2012 and 28% in 2023), and broader legislative developments—such as the implementation of a national smoking ban in Czech healthcare facilities in 2017—limit direct attribution of these trends solely to the hospital’s specific HPH activities. Furthermore, a notable rise in the use of alternative tobacco and nicotine products, particularly among nurses (increasing from 0.1% to 11.0%), underscores continuing workforce-directed prevention and cessation needs [13].

Occupational health and workplace safety constituted another substantial component of the evidence [30,39]. In a nationwide Taiwanese survey, Wei et al. [39] found a high prevalence of workplace violence among nurses. Ordinary HPH certification was not associated with a lower overall risk of workplace violence; however, employment in hospitals characterized as outstanding HPHs was associated with lower risks for selected violence outcomes. These findings suggest a possible relationship between more advanced organizational health-promotion performance and workplace safety, but the cross-sectional design does not permit causal interpretation [39]. Chiou et al. [30] likewise documented substantial variation in nurses’ health and occupational hazards according to clinical work setting. Differences were observed in general health, depressed mood, musculoskeletal problems, injuries, harassment, and violence, highlighting the importance of tailoring workplace health promotion to specific occupational environments rather than treating the healthcare workforce as a homogeneous population [30].

Abedi et al. [14] provided a more intervention-oriented contribution through a best-practice implementation project addressing violence management among nurses in an HPH setting. Initial assessment identified limited baseline compliance with several recommended practices, including staff training (49%), timely support following violent incidents (8%), and organizational safe-environment policies (22%). Following a multifaceted implementation strategy involving training, communication-skills development, audit and feedback, guideline implementation, and organizational safety measures, significant improvements were reported in evidence-based violence-management practices and staff awareness. Importantly, however, the intervention was based on Joanna Briggs Institute (JBI) best-practice criteria for workplace-violence management rather than being an intervention derived directly from HPH standards. It therefore provides evidence for the feasibility of workforce-oriented interventions within an HPH setting rather than evidence of a direct HPH-specific treatment effect [14].

A further group of studies examined the relationship between workforce health and organizational conditions [28,45]. Whitehead et al. [28], using cross-sectional structural equation modeling among 600 specialized hospital nurses, identified significant relationships among lifestyle, quality of life, organizational culture, and job satisfaction. Specifically, health-promoting lifestyle dimensions—such as spiritual health, physical activity, and stress management—were strong predictors of physical and psychological quality of life, which in turn influenced organizational culture, while interpersonal communication directly predicted job satisfaction. These findings support a multidimensional representation of workforce health in which individual behaviors and organizational conditions are interrelated, although the cross-sectional design again prevents causal interpretation [28]. Similarly, Bilterys and Dedobbeleer [45] demonstrated that the implementation of a health-promoting workplace for nurses is shaped by its compatibility with existing organizational practices. While they reported high compatibility with learning systems, workplace safety, and lifestyle support, nurse participation in decision-making relative to their work environment was entirely absent due to rigid, lingering bureaucratic structures, further highlighting the crucial link between organizational conditions and workforce well-being [45].

Organizational assessments also showed that the workforce component of health promotion extended beyond individual health outcomes [45,47]. Bilterys and Dedobbeleer [45] identified strengths in organizational learning, healthy and safe working conditions, and support for healthy lifestyles, alongside weaker policy integration and limited participation of nurses in organizational decision-making. Comparing Estonian HPH and non-HPH hospitals, Põlluste et al. [47] found widespread workplace risk assessment among HPH hospitals and more frequent use of staff job-satisfaction surveys in HPH than in non-HPH organizations. These indicators describe organizational practices relevant to workforce health but should not be interpreted as direct evidence of improved employee health [45,47].

At the organizational and network level, Lee et al. [33] reported perceived development of workplace-health-promotion capacity among Taiwanese HPH hospitals, including changes in organizational strategy, resource allocation, staff participation, and provision of staff-oriented health services. Huang et al. [32] provided a broader account of the Taiwanese HPH experience, integrating evidence concerning staff health behaviors, participation, workplace safety, and organizational health-promotion activities. Because this report synthesized evidence from the Taiwanese HPH program, including findings related to studies represented elsewhere in the corpus, it was treated as contextual evidence rather than as an additional independent estimate of workforce outcomes [32].

The strongest controlled evidence within this domain was provided by Svane et al. [55], who conducted a randomized implementation trial across 36 clinical departments involving 2529 staff members and more than 5000 patients. The structured WHO-HPH implementation program significantly improved several measures of organizational implementation, including the identification of lifestyle risks, delivery of health-promotion interventions, and compliance with WHO-HPH standards. However, these structural and procedural improvements were not accompanied by detectable differences in staff health status. This finding is particularly relevant because it demonstrates that successful implementation of HPH structures and practices should not be assumed to produce measurable short-term improvements in workforce health [55].

Overall, the 14 reports demonstrate that workforce-oriented HPH activity encompasses considerably more than individual lifestyle promotion. The literature addressed health behaviors and preventive practices, occupational hazards and workplace violence, psychological and quality-of-life indicators, job satisfaction, organizational culture, staff participation, workplace risk assessment, and organizational capacity for workforce health promotion. Although several studies identified favorable associations between HPH environments, participation in health-promotion activities, and selected workforce outcomes, the predominance of cross-sectional and self-reported evidence, heterogeneity of outcomes, overlapping evidence from some national HPH programs, and scarcity of controlled studies substantially limit causal conclusions. The available evidence therefore supports the relevance and organizational feasibility of workforce health promotion within HPH&HS, while evidence that HPH implementation itself improves workforce health outcomes remains limited.

### 3.5. Organizational Conditions Shaping Implementation

The 17 reports primarily classified within the implementation conditions domain showed that implementation of the HPH&HS model was shaped by the interaction of organizational, professional, and contextual conditions rather than by the adoption of individual health-promotion activities alone [19,21,34,35,36,37,38,40,41,43,44,46,48,49,54,56,58]. Across the studies, recurrent determinants concerned leadership and strategic commitment, organizational structures and resources, workforce participation and competencies, integration into routine practice, and the wider policy environment [19,21,34,35,37,38,41,43,44,46,48,49,54,56]. Because several publications originated from related HPH programs—particularly the Taiwanese and South African experiences—recurring findings within these clusters were interpreted as complementary evidence rather than as independent replications [34,35,36,37,38,40,41].

Leadership and explicit organizational commitment emerged as central implementation conditions. Studies conducted in different healthcare systems described management support as important for legitimizing health promotion, allocating organizational attention and resources, and incorporating HPH objectives into institutional priorities [19,34,35,37,41,43,44,46,48,56]. In the Taiwanese HPH network, support from hospital superintendents was the most frequently reported facilitator, followed by support from unit or department directors and the incorporation of health promotion into hospital mission and development goals [34]. Conversely, insufficient managerial commitment or failure to integrate health promotion into organizational priorities was described as an obstacle in other contexts [19,21,37,48,49]. These findings indicate that leadership operated not only as formal endorsement but also through its capacity to translate health promotion into organizational goals, policies, and implementation structures [19,34,35,37,46,48].

Dedicated organizational structures were another recurrent enabling condition. Health-promotion committees, coordinators, defined responsibilities, and mechanisms for cross-departmental coordination were associated with greater implementation capacity [34,38]. In Taiwan, 45 of 52 HPHs had established health-promotion committees, and the presence and effective functioning of these committees predicted fewer perceived implementation barriers. Coordinators also played a pivotal role: those who convened meetings, devoted more time to health promotion, or were directly involved in compiling program outcomes tended to perceive fewer barriers or more facilitators [34]. At a broader organizational level, Lin and Lin [38] similarly conceptualized health-promotion effectiveness in relation to institutional structures such as hospital-level committees, annual policies and plans, dedicated budgets, staff training, and the scope of health-promotion programs.

The availability of resources strongly conditioned whether organizational commitment could be translated into practice priorities [19,34,35,36,37,40,41,44,48]. Financial support, dedicated personnel, protected time, infrastructure, and access to relevant expertise were recurrent implementation requirements, whereas insufficient funding, staff shortages, workload, and competing clinical responsibilities constrained implementation [19,21,34,37,40,41,44,48]. In Taiwan, access to resources was among the most frequently reported organizational facilitators, while government project funding also represented an important external enabler [34]. The qualitative evidence from Ghana similarly emphasized management commitment together with resource allocation as necessary conditions for creating an organizational environment capable of sustaining health-promotion activities [48].

Workforce participation represented a particularly important but unevenly developed component of implementation [19,21,34,35,48,49,54]. The included studies described staff engagement not simply as individual compliance with HPH activities but as participation in organizational change [19,35,37,48]. In the Taiwanese network, opportunities for staff to undertake health-promotion activities, on-the-job training, involvement of staff already motivated towards health promotion, and inclusion of health promotion within merit systems were identified as facilitators. At the same time, staff detachment, lack of interest among some medical professionals, absence of incentives, and resistance to change were among the most frequently reported barriers. Notably, staff participation remained one of the least developed dimensions of organizational capacity despite improvements in organizational structures [34,35]. This pattern illustrates a distinction between establishing formal HPH infrastructure and achieving engagement among frontline professionals [19,34,48].

Knowledge, competences, and professional learning were closely related to participation. Several reports indicated that implementation required staff to understand health promotion beyond conventional curative roles and to acquire appropriate knowledge, communication skills, and implementation competences [19,34,40,41,43,44,48,56]. Training therefore appeared both as a means of developing competence and as a mechanism for supporting organizational change [19,37,48]. In the WHO-HPH Recognition Process, staff and managers described implementation as promoting awareness, learning, cultural reorientation, normalization and legitimacy, and a more structured and evidence-based approach to health promotion. However, they also identified time requirements, the need for advance training, translation, and adaptation to local circumstances as areas requiring improvement [54].

A recurrent challenge was the integration of health promotion into routine clinical and organizational work [19,48,54]. Several reports described tensions between health-promotion objectives and established hospital priorities, particularly when health promotion was perceived as additional work rather than as part of core organizational practice [19,35,40,41,48,56]. Hazavehei et al. [19], for example, documented staff concerns that implementing HPH standards could increase duties and reduce accuracy in personnel performance, alongside recommendations for improved management and interpersonal communication. Similarly, the Taiwanese experience showed that understaffing and strong inter-hospital competition could make it difficult to devote resources to activities perceived as peripheral to the hospital’s core mission [35]. These findings suggest that implementation was facilitated when health promotion was normalized within existing roles, procedures, and organizational systems rather than superimposed as an additional set of tasks [19,48,54].

The studies also showed that implementation was influenced by conditions outside individual healthcare organizations. Government policy, financing and reimbursement arrangements, accreditation systems, professional and academic networks, and access to community resources could either support or constrain HPH development [34,35,36,37,43,44,48]. In Taiwan, inadequate national health-insurance coverage for health promotion and incoherent government policies were among the most frequently reported barriers, whereas government project funding, incorporation of health promotion into hospital accreditation, and access to community resources were prominent facilitators [34]. Thus, organizational capacity was partly contingent on an external environment that could provide legitimacy, incentives, resources, and institutional alignment [34,35,36,37,43,44,48].

Across the corpus, organizational capacity consequently emerged as a multidimensional construct rather than a single implementation characteristic [21,34,35,38,41,48,49]. The Taiwanese studies operationalized this capacity through dimensions including mission and strategy, leadership, organizational culture, structure, systems, management practices, and staff participation [34,35]. Lin and Lin [38] similarly found that the health-promoting status of hospitals encompassed policy and leadership, physical environment, healthy culture, and health resources and activities, linking these organizational characteristics to the effectiveness of health-promotion implementation. Evidence from other national contexts likewise indicated that implementation depended on combinations of organizational commitment, infrastructure, human resources, competences, communication, and integration rather than on any single determinant [21,48,49].

Taken together, the 17 reports indicate that implementation of the HPH&HS model is shaped by the degree to which health promotion becomes organizationally supported, adequately resourced, professionally shared, and integrated into routine practice. Leadership and formal structures can provide the conditions for implementation, but they do not by themselves ensure workforce engagement or operational integration. Conversely, insufficient resources, competing workloads, limited staff participation, weak competencies, resistance to change, and misalignment with external policy or financing systems can restrict implementation even where formal organizational commitment exists. The evidence therefore portrays HPH&HS implementation as a multidimensional organizational process in which leadership, structures, resources, workforce participation, competencies, routine work processes, and the external institutional environment interact in shaping the depth and sustainability of organizational change.

## 4. Organizational Case Example: Workforce Health Promotion Within ASUFC

To complement the narrative synthesis, the experience of Friuli Centrale Healthcare University Trust (ASUFC), Italy, is presented as an organizational case example. The case is not part of the literature corpus and is not intended as independent evidence of the effectiveness of the HPH&HS model. Rather, it illustrates how workforce health promotion can be operationalized within a healthcare organization through the coexistence of governance structures, organizational programs, and individual-level services within a common HPH&HS framework.

ASUFC is a complex and geographically distributed healthcare system integrating hospital, territorial, preventive, rehabilitative, and mental health services across the region. The organization employs approximately 9000 healthcare and administrative professionals, including physicians, nurses, psychologists, allied health professionals, and support staff. Within this context, workforce well-being and health-promotion initiatives are situated within the regional HPH&HS network and the activities of the local ASUFC HPH Committee.

At the institutional level, HPH&HS activities are coordinated through the ASUFC HPH Committee, chaired by a dedicated coordinator working in collaboration with Strategic Management. The committee is composed of multidisciplinary referents representing clinical and territorial departments, human resources, and occupational health services. This internal network is responsible for translating the HPH framework into local strategic planning, organizational development, and periodic self-assessment processes.

The HPH&HS governance structure operates across the following interconnected levels:

- Regional Policy and Strategic Mandate. The overarching governance is provided by the Central Health Directorate, Social Policies and Disabilities, which integrates HPH strategies into the regional health management guidelines.
- The Regional HPH Coordinator, based within the Azienda Regionale di Coordinamento per la Salute (ARCS), supports the regional coordination of HPH&HS activities and their consistency with the principles of the international HPH movement, including the Ottawa Charter and the Budapest and Vienna Declarations [59,60,61].
- Local Institutional Coordination. At the institutional level, implementation is managed by HPH local coordinators. Within ASUFC, this role is assigned to a single local coordinator who works in collaboration with Strategic Management to support the translation of regional HPH&HS objectives into local organizational activities.
- Regional HPH&HS activities also involve designated contact persons and specialized working groups that support coordination with other regional health-promotion programs. These structures provide opportunities for collaboration across programs and organizational areas.
- Link Professionals for Health Promotion. To support the implementation of health-promotion activities in clinical and community settings, ASUFC identified dedicated link professionals within key organizational areas. Their role is to facilitate communication and the dissemination of HPH&HS principles across departments and professional settings.

Within this organizational framework, workforce health promotion has been pursued through different initiatives developed and evaluated independently. These include the Mindfulness-Oriented Professional Resilience (MOPR) program, a structured group intervention aimed at strengthening professional resilience through psychoeducation, mindfulness, reflective work, and physiological self-regulation, and Aver Cura di Chi Ci Cura (‘Caring for Those Who Care’), a workforce health-promotion service provided within ASUFC that offers employees accessible multi-professional assessment, lifestyle counseling, and self-regulation support. Both initiatives have been evaluated separately. Previous evaluations of MOPR have examined outcomes related to professional resilience, whereas available evaluations of Aver Cura di Chi Ci Cura have focused on selected workforce health and well-being indicators [62,63,64].

Importantly, these initiatives should not be interpreted as components of a formally integrated or empirically tested multi-level intervention model. They were developed for different purposes, target populations, and organizational contexts and have been evaluated independently. Their relevance to the present review lies instead in their coexistence within a common HPH&HS governance framework, in which different workforce-oriented activities are made available within the same organizational context. The ASUFC experience therefore provides a practice-based illustration of how workforce-oriented activities can be situated within organizational structures informed by the HPH&HS framework, while the effectiveness of the individual programs and services remains dependent on their respective evaluations. As an organizational case example, it also highlights the distinction between embedding workforce health promotion within an organization and demonstrating measurable improvements in workforce health, a distinction that is considered further in Section 5.

## 5. Discussion

The present narrative review examined healthcare workforce health and well-being through the specific lens of the HPH&HS framework, bringing together 51 reports spanning more than 25 years of conceptual development, standards-based assessment, organizational implementation, and workforce-related research. Across the four domains examined in this review, a coherent pattern emerges. Workforce health is conceptually embedded within HPH&HS; it has become increasingly formalized through standards, governance structures, and organizational processes; a range of workforce-focused interventions and outcomes has been investigated; and implementation appears to depend on organizational conditions including leadership, resources, staff participation, competencies, and integration into routine practice. However, these dimensions are not equally supported by empirical evidence, with considerably stronger evidence concerning organizational implementation than direct and sustained workforce health outcomes.

The synthesis indicates that workforce health is not a peripheral concern within HPH&HS, but is embedded in its conceptual foundations and subsequently formalized through standards and organizational requirements [2,5]. At the same time, the available literature reveals an important gap between the recognition of workforce health as an organizational responsibility and the evidence demonstrating that HPH&HS implementation produces measurable improvements in employee health and well-being [4].

The literature can be interpreted as reflecting a gradual shift from early settings-based conceptualizations of healthcare organizations towards increasingly structured mechanisms for incorporating health promotion into organizational practice. The development of standards, self-assessment procedures, governance structures, and links with quality-management systems provided formal mechanisms through which health promotion could be connected with organizational processes [65]. From this perspective, the HPH&HS literature increasingly moves beyond isolated health-promotion projects towards consideration of organizational capacity for health promotion [3]. Workforce health occupies an important position within this development because healthcare professionals are both a population addressed by health-promotion activities and agents involved in their implementation [3,66].

Crucially, the synthesized literature indicates that organizational institutionalization and direct workforce health improvement represent related but conceptually and empirically distinct levels of outcome [5]. To date, the preponderance of HPH&HS evaluative research has focused on the former, assessing parameters such as compliance with quality standards, organizational readiness, implementation capacity, and the establishment of dedicated health-promoting infrastructures [65]. Conversely, robust empirical evidence demonstrating direct, measurable changes in employee health status remains exceptionally scarce, with the existing literature heavily reliant on cross-sectional designs, subjective self-reported measures, and observational comparisons [4]. The department-level randomized controlled trial by Svane et al. [54] is particularly informative in this respect: although structured HPH implementation improved organizational and procedural indicators, these changes were not accompanied by detectable differences in staff health outcomes. This finding illustrates why organizational implementation and workforce health outcomes should be evaluated as related but distinct dimensions of HPH&HS [54]. Rather than questioning the relevance of workforce health within HPH&HS, this finding underscores the importance of distinguishing the implementation of health-promoting organizational conditions from evidence of measurable changes in workforce health [5,55].

This implementation–outcome distinction also provides a useful perspective for interpreting the organizational conditions identified across the reviewed literature. Specifically, factors such as high-level leadership commitment, dedicated administrative structures (such as specialized health-promotion committees and coordinators), the allocation of earmarked resources, active frontline participation, tailored professional competencies, and the strategic alignment of hospital activities with external health policy and reimbursement systems were repeatedly identified as conditions associated with HPH&HS implementation [34,67]. Crucially, these systemic prerequisites should not be conceptualized as direct, standalone workforce health interventions; rather, they can instead be understood as elements of the organizational infrastructure within which workforce health promotion may be implemented [7,67]. Conversely, the nominal or formal adoption of HPH standards without sufficient funding, frontline ownership, protected time, or integration into routine clinical pathways risks producing mere symbolic compliance at the level of administrative structures and documentation. Formal adoption of standards should therefore not be assumed to produce corresponding changes in employees’ everyday working conditions or health [34].

### 5.1. From Health-Promotion Projects to Health-Promoting Organizations

A key implication of this synthesis is that workforce health promotion within the HPH&HS framework extends beyond the provision of discrete wellness activities or individual lifestyle-focused interventions. Across the conceptual, standards-based, and empirical literature, a recurrent distinction emerges between implementing isolated health-promotion projects and developing longer-term organizational capacity to incorporate health promotion into routine structures, processes, and organizational culture [3,67]. This distinction is consistent with the settings-based perspective underlying the Ottawa Charter and HPH&HS, in which the healthcare organization is understood not simply as a setting in which interventions are delivered, but as an organizational environment whose structures, culture, and working conditions may influence the health and well-being of its workforce [59].

The literature reviewed here can be interpreted as reflecting a gradual shift in this direction. Early conceptual contributions questioned project-based approaches in which health promotion remained separate from the core organizational and clinical functions of healthcare institutions [5,12]. Subsequently, HPH standards and self-assessment frameworks provided formal mechanisms for incorporating health promotion into organizational policy, workplace conditions, staff participation, and quality-management processes [65]. Implementation research has further examined organizational capacity through factors including leadership, dedicated structures, resource allocation, professional competencies, monitoring, workforce participation, and the incorporation of health promotion into routine practice [34,67]. Taken together, these developments suggest that HPH&HS institutionalization involves incorporating health promotion into governance, organizational structures, routine processes, and evaluation rather than limiting it to discrete or temporary activities [3,67].

This conceptual distinction is exceptionally salient when applied specifically to healthcare workforce health. Employee-directed initiatives targeting physical activity, healthy nutrition, tobacco cessation, or individual stress-coping strategies may form part of workplace health promotion, but their presence alone does not indicate that health promotion has been incorporated into broader organizational structures and processes [3]. The literature reviewed here consistently identifies working conditions, organizational culture, staff participation, leadership, workload, professional development, and the physical and psychosocial work environment as relevant dimensions of workforce health within the HPH&HS framework [38]. Accordingly, a settings-based approach to workforce health places attention not only on individual health behaviors and coping strategies but also on the institutional, environmental, and managerial conditions associated with employee health and well-being [38,68].

At the same time, an organizational settings perspective does not exclude individually targeted workforce interventions. Rather, the literature includes both organizational approaches addressing working conditions and structures and employee-directed initiatives addressing individual health behaviors, coping, and self-regulation [8]. Healthcare organizations can systematically optimize macro-level working conditions and organizational structures while simultaneously providing accessible, targeted resources to cultivate employees’ personal coping strategies, self-regulatory mechanisms, and health-promoting behaviors [68]. From a salutogenic perspective, organizational and individual resources can be considered conceptually relevant to workforce health; however, the reviewed literature does not establish how these levels interact or whether their combination produces greater health benefits than either approach alone [7,68]. The distinction between individual- and organizational-level approaches should therefore not be interpreted as requiring an exclusive focus on either level. A more relevant question is how workforce-oriented initiatives are situated within broader organizational policies, structures, working conditions, and health-promotion processes [8,9].

The implementation literature further indicates that formal adoption of HPH standards alone is insufficient to characterize the extent to which health promotion has been incorporated into organizational practice [5,35]. Leadership commitment was repeatedly identified alongside other implementation conditions, including adequate resources, dedicated organizational structures, staff competencies, and opportunities for workforce participation [36,67]. Conversely, implementation may be constrained when health promotion is perceived as additional or peripheral work or when frontline professionals have limited involvement in decision-making processes [48,51]. Competing clinical priorities and resource constraints, particularly limited time, personnel, and dedicated funding, were also recurrent barriers to implementation [46,69]. Taken together, these findings suggest that HPH&HS institutionalization is better understood in terms of the extent to which health promotion is incorporated into governance, professional roles, routine practice, and organizational culture rather than solely through certification or formal designation [3,35,67].

Evaluation of workforce health promotion within HPH&HS may therefore benefit from considering not only the activities offered to employees but also the extent to which health promotion is reflected in organizational policies, governance structures, working conditions, and routine practice [3,67]. The movement from isolated projects towards broader organizational capacity is therefore a central feature of the settings-based interpretation of health promotion reflected in the HPH&HS literature [3,68]. An important empirical question nevertheless remains: whether greater organizational institutionalization of HPH&HS is associated with measurable and sustained improvements in workforce health and well-being [4,55].

### 5.2. The Workforce as Both Target and Agent of Health Promotion

A distinctive feature emerging from this synthesis is the dual position occupied by healthcare professionals within the HPH&HS framework. The workforce is both a target population whose health, safety, and psychosocial working conditions are addressed within HPH&HS and an important agent through whom health promotion is implemented in healthcare practice [2,9]. This dual role, already evident in the foundational HPH literature, distinguishes the HPH&HS perspective from approaches in which workforce health is considered primarily as a separate employee-health or occupational-health objective [3,9]. Within HPH&HS, staff well-being is therefore considered not only as an occupational-health objective but also in relation to the organization’s broader capacity to implement health promotion [31,66].

This relationship is relevant to implementation because healthcare professionals contribute to translating organizational health-promotion principles into everyday practice [35]. Consistent with this interpretation, the reviewed literature identifies staff knowledge, competencies, participation, and professional engagement as relevant components of organizational capacity for health promotion [38,67]. Conversely, limited awareness of HPH principles, insufficient training, lack of protected time, heavy workloads, and perceptions of health promotion as additional work emerged as recurrent barriers to implementation [48,51]. Healthcare professionals should therefore not be considered solely as recipients of workforce health-promotion policies; their participation and engagement also form part of the organizational processes through which health promotion is implemented in practice [9,68].

At the same time, emphasizing staff agency should not shift responsibility for workforce health promotion from the organization to individual healthcare professionals [12,70]. Consistent with the settings-based approach, healthcare professionals’ capacity to participate in health promotion is influenced by the organizational environment in which they work [2,68]. The reviewed literature indicates that workforce participation and engagement are associated with organizational conditions including leadership, organizational culture, staffing and workload, resource availability, participatory decision-making, and the integration of health promotion into routine practice [35,48,67]. This suggests a potential tension within HPH&HS when healthcare professionals are expected to contribute to health promotion without corresponding organizational conditions supporting their own health and participation [44,70].

This dual position of the workforce suggests a potentially reciprocal relationship between workforce health and organizational health-promotion capacity. Supportive organizational conditions may provide resources that enable healthcare professionals to maintain their health and participate in health-promoting practices, while an adequately supported and engaged workforce may contribute to the organization’s capacity to implement health promotion [38,68]. The reviewed literature provides conceptual and observational support for associations among staff participation, organizational culture, professional competencies, and organizational health-promotion capacity [38,67]. However, the available evidence does not establish the direction or magnitude of these relationships. These relationships should therefore be regarded as plausible organizational mechanisms rather than as demonstrated causal pathways [67,68].

This conceptual distinction has practical implications for the positioning of workforce health within HPH&HS frameworks. Within a settings-based perspective, employee-directed initiatives can be understood as one component of a broader organizational responsibility for creating conditions that support workforce health and participation [9,68]. Within this systemic perspective, establishing an environment that preserves healthcare professionals’ health and empowers them to execute their health-promoting roles is a fundamental organizational duty [2]. The findings also suggest that workforce participation may extend beyond attendance at health-promotion activities to include involvement in needs assessment, intervention development, evaluation of working conditions, and organizational decision-making [12,18,52].

Recognizing healthcare professionals as both targets and agents of health promotion supports the inclusion of workforce health within broader organizational health-promotion capacity rather than treating it solely as a separate employee-oriented program [3,9]. This dual positioning also highlights the importance of evaluating not only whether health-promotion structures and activities are implemented, but also whether healthcare professionals have access to the resources, competencies, and opportunities for participation needed to engage with them [48,67]. Workforce health and organizational health-promotion capacity can therefore be considered related dimensions of HPH&HS, although the empirical relationships between them require more robust longitudinal and intervention research [4,29].

### 5.3. From the Literature to the ASUFC Organizational Experience

The organizational experience of the Azienda Sanitaria Universitaria Friuli Centrale (ASUFC) provides a practice-based context in which several of the structural and conceptual themes identified across this narrative review can be illustrated. The case is considered separately from the review corpus and is used to illustrate how selected themes emerging from the literature can be observed within a specific organizational context. Specifically, the ASUFC case illustrates how workforce-oriented initiatives with distinct purposes and modes of delivery can coexist within an organizational governance context informed by HPH&HS principles [64]. This organizational coexistence is relevant to the present review because the HPH&HS literature emphasizes the importance of situating workforce-oriented activities within broader organizational structures, governance processes, professional participation, and routine practice [3,66].

Several structural characteristics of the ASUFC experience are consistent with organizational conditions identified across the HPH&HS literature. Specifically, regional and local coordination structures, the multidisciplinary HPH committee, links with executive management, and the appointment of link professionals reflect organizational features that are also emphasized in the literature, including leadership support, dedicated structures, and cross-organizational coordination [64,67]. Furthermore, the linkage between regional governance and local organizational activities illustrates how external policy and coordination structures can be connected with local HPH&HS implementation [34]. These structural similarities should not be interpreted as evidence of HPH&HS effectiveness; rather, they indicate that the ASUFC governance context includes several organizational features also identified in the reviewed literature as relevant to implementation [34,67].

Within this governance context, the Mindfulness-Oriented Professional Resilience (MOPR) training and the ‘Aver Cura di Chi Ci Cura’ program represent two distinct workforce-oriented initiatives that were developed and evaluated independently. The MOPR program is a structured educational intervention addressing professional resilience through psychoeducation, mindfulness meditation, Acceptance and Commitment Therapy (ACT)-informed reflective work, and autonomic self-regulation techniques to help staff manage occupational stressors and compassion fatigue [62,63]. On the other hand, “Aver Cura di Chi Ci Cura” operates as an accessible, multi-professional health-promotion service; it proactively addresses employee health by utilizing non-invasive electromedical technologies to assess allostatic load and Medically Unexplained Symptoms (MUS), while simultaneously offering personalized lifestyle counseling, circadian rhythm restoration, and cardiac coherence biofeedback [64]. The two initiatives differ in purpose and mode of delivery and should not be interpreted as components of a combined intervention model. Their relevance here lies in their availability within the same broader organizational context [8,64,68].

However, the ASUFC experience also illustrates why a conceptual and evaluative distinction between systemic organizational embedding and empirically demonstrated effectiveness remains paramount [5,55]. Although both MOPR and Aver Cura di Chi Ci Cura coexist under the same regional HPH&HS governance umbrella, they were developed to address highly distinct salutogenic objectives and have been evaluated through separate, independent methodologies [63,64]. Their parallel institutional availability does not, in itself, constitute evidence of a combined, synergistic clinical effect, nor does it establish that their coexistence within the same organizational context has produced measurable organization-wide improvements in workforce health. In this regard, the ASUFC case directly mirrors a persistent methodological limitation widely documented in the international settings-based literature: healthcare institutions are often highly successful in establishing administrative structures, strategic programs, and dedicated wellness services aligned with health-promoting principles, yet they frequently lack the robust, longitudinal, and controlled evidence required to causally link the overall degree of organizational institutionalization to direct, objective changes in workforce health outcomes [4,55].

This methodological distinction suggests an important direction for future evaluation for both the ASUFC experience and, more broadly, for evaluative research within HPH&HS organizations globally [5,38]. Specifically, future evaluations should move beyond merely documenting the compliance or short-term effectiveness of isolated, individual initiatives toward systematically examining the longitudinal relationship between whole-system organizational health-promotion capacity and workforce health outcomes over time [67,68]. Such an approach would benefit from the parallel assessment of validated organizational indicators—such as strategic leadership commitment, active staff participation, the availability and accessibility of salutogenic resources, and the formal integration of healthy-workplace standards—alongside direct, individual workforce health and well-being outcomes [38,65]. Ultimately, robust longitudinal and intervention-based designs are particularly valuable for examining the temporal relationships and potential causal pathways of these dynamics—specifically, whether progressive improvements in systemic organizational capacity actually precede, accompany, or occur independently of measurable, long-term enhancements in employee health, resilience, and professional quality of life [29,55].

Viewed in this way, the ASUFC case is best understood not as a validated model to be generalized to other healthcare systems, but as a practice-based illustration of the coexistence of HPH&HS governance structures and independently developed workforce-health initiatives within one healthcare organization. Its contribution to the present paper is therefore illustrative and hypothesis-generating rather than evidentiary. The case highlights, in an organizational setting, the distinction emerging from the review between embedding workforce health promotion within governance and organizational structures and demonstrating measurable improvements in workforce health. Establishing whether, how, and under what conditions these organizational features are associated with sustainable workforce outcomes remains an important priority for future HPH&HS research [62,64].

### 5.4. Limitations

The present review has several strengths. It applies a workforce-centered analytical lens to a literature that has traditionally examined healthcare professionals alongside other HPH&HS target populations and organizational objectives. The structured search across four major databases, explicit eligibility criteria requiring both an identifiable HPH/HPH&HS framework and a substantive workforce component, full-text assessment, and standardized data extraction increased the transparency of the review process. In addition, distinguishing organizational and implementation indicators from direct workforce health outcomes allowed the synthesis to avoid assuming that successful implementation necessarily translates into improved employee health. Finally, potentially overlapping publications were identified and interpreted as related evidence rather than treated uncritically as independent confirmation of the same findings.

Several limitations should nevertheless be considered. First, the review was designed as a narrative synthesis rather than a systematic review or meta-analysis. Although a structured search and explicit selection procedures were used, the heterogeneity of the literature in terms of study design, populations, organizational settings, HPH standards, interventions, and outcomes precluded quantitative synthesis. The four primary narrative domains used to organize the evidence should therefore be understood as analytical categories developed for the purposes of this review rather than as an established taxonomy of HPH&HS workforce research. Individual reports frequently addressed more than one domain, even though each was assigned to a single primary domain to support a coherent synthesis.

Second, the characteristics of the available evidence limit causal inference. Cross-sectional and self-assessment designs represented approximately half of the included literature, while controlled and longitudinal studies directly examining workforce health outcomes were uncommon. Many studies assessed implementation, organizational readiness, compliance with standards, perceived barriers, or health-promotion activities rather than changes in employee health itself. Consequently, the literature provides considerably stronger evidence regarding how workforce health promotion is conceptualized and implemented within HPH&HS than regarding its effectiveness in improving workforce health and well-being.

Third, the geographical distribution and structure of the literature require caution. A substantial proportion of reports originated from Iran and Taiwan, and several publications within particular national contexts were related through common HPH networks, programs, or datasets. Although these reports provided valuable information on implementation and organizational development, they cannot be regarded as fully independent replications. Conversely, evidence from several healthcare systems and world regions remains limited. Differences in healthcare governance, financing, workforce organization, occupational health regulation, and the maturity of national HPH networks may also constrain the transferability of findings between settings.

Additional limitations arise from the search and evidence-selection process. Six reports sought for full-text retrieval could not be obtained and therefore could not undergo final eligibility assessment. Moreover, despite the use of broad workforce-related search terms, relevant HPH&HS publications may have been missed when workforce issues were not represented in titles, abstracts, or indexed terms. The inclusion of conceptual papers, reviews, implementation studies, and empirical investigations was appropriate to the broad organizational question addressed here, but it also increased methodological heterogeneity. In addition, study selection was conducted by a single reviewer, and reviewer-dependent selection bias therefore cannot be excluded, although uncertain and borderline records were re-examined against the predefined eligibility criteria. No formal risk-of-bias assessment was undertaken across this diverse corpus; accordingly, the synthesis was interpretive and greater weight was placed on convergence across independent settings and study designs rather than on numerical aggregation of findings. Consequently, the conclusions of the review concern patterns, consistencies, and evidence gaps rather than graded estimates of intervention effects.

Finally, the ASUFC organizational experience should be interpreted within the limits of a descriptive case example. It was deliberately kept separate from the review corpus and was included to illustrate how workforce-oriented initiatives can coexist within an HPH&HS governance context. The initiatives described were developed and evaluated independently, and the case does not provide evidence for the effectiveness of an integrated organizational model or for organization-wide improvements in workforce health. Its value in the present paper is therefore primarily illustrative and hypothesis-generating.

### 5.5. Future Directions

The findings identify several priorities for future HPH&HS workforce research. Most importantly, future studies should move beyond assessing whether health-promotion structures and activities have been implemented and examine whether, how, and under what conditions organizational implementation translates into measurable improvements in workforce health. This requires a clearer distinction between implementation outcomes, such as adoption of standards, organizational capacity, participation, and program reach; organizational or work-environment indicators, such as changes in working conditions, organizational culture, or access to health-promoting resources; and workforce health outcomes, including physical and psychological health, well-being, occupational stress, work ability, and other relevant indicators.

Longitudinal and controlled designs would substantially strengthen this evidence base. In particular, repeated assessment of both organizational conditions and workforce outcomes could help determine whether improvements in organizational health-promotion capacity precede changes in employee health and whether these relationships are sustained over time. Where feasible, comparisons between organizations or organizational units with different degrees of HPH&HS implementation could complement evaluations of specific interventions. Such research would also allow potential mechanisms—including leadership, employee participation, organizational culture, resource availability, and integration into routine practice—to be examined rather than treated only as contextual implementation factors.

Greater standardization of workforce-related indicators would also facilitate comparison across HPH&HS settings. The existing literature employs heterogeneous measures ranging from compliance with workplace standards and health behaviors to job satisfaction, stress, workplace violence, and general health. Developing a core set of workforce indicators aligned with contemporary HPH&HS standards could help distinguish organizational processes from health outcomes and support longitudinal benchmarking across organizations and national networks. At the same time, standardization should allow sufficient flexibility to account for differences among professional groups, organizational settings, and healthcare systems.

Future research should also broaden the geographical base of the evidence and examine the sustainability and equity of workforce health promotion. Greater representation of healthcare systems currently underrepresented in the literature would help clarify which organizational mechanisms are transferable across contexts and which depend on specific governance or policy arrangements. Within organizations, attention should be paid to whether health-promoting resources are equally accessible to different occupational groups, work locations, employment arrangements, and shift patterns, and whether implementation remains sustainable when initial project funding or leadership support changes.

Finally, the distinction between individual interventions and organizational health promotion deserves further empirical investigation. Rather than assuming a predetermined multi-level intervention model, future studies could examine whether the outcomes of employee-directed programs vary according to the organizational conditions in which they are implemented.

For ASUFC, this perspective suggests a future evaluation strategy in which the effectiveness of individual initiatives continues to be assessed separately while organization-level indicators of workforce health promotion are monitored in parallel. Over time, such an approach could provide the basis for investigating whether changes in organization-level HPH&HS indicators are associated with changes in workforce health and well-being across the organization. More broadly, developing this type of evidence would allow HPH&HS research to move from demonstrating that workforce health belongs within the health-promoting organization to determining which organizational strategies and conditions are associated with better workforce health outcomes, for whom, and under what circumstances.

## 6. Conclusions

This narrative review indicates that healthcare workforce health and well-being are not peripheral concerns within the Health Promoting Hospitals and Health Services framework, but an intrinsic component of its conceptual foundations, standards, and organizational development. Across more than 25 years of literature, workforce health has become increasingly formalized through HPH&HS standards, governance structures, and implementation mechanisms through which workforce health promotion can be incorporated into organizational practice. However, the evidence supporting organizational implementation is substantially more developed than the evidence demonstrating direct and sustained improvements in workforce health outcomes.

The findings therefore highlight an important distinction between embedding workforce health promotion within a healthcare organization and demonstrating that such organizational development improves workforce health. The reviewed literature documents the inclusion of working conditions, staff participation, healthy lifestyles, occupational risks, and psychosocial well-being within HPH&HS, but heterogeneous outcomes, predominantly cross-sectional designs, and limited controlled and longitudinal evidence preclude strong conclusions regarding effectiveness.

The ASUFC organizational experience illustrates how independently developed workforce-oriented programs and services can coexist within a common HPH&HS governance framework, without implying that they constitute a formally integrated or empirically validated intervention model. Future research should build on this organizational perspective by linking implementation processes, organizational conditions, and clearly defined workforce health outcomes through longitudinal and controlled designs. Such evidence is needed to move HPH&HS research from establishing workforce health as a core organizational responsibility towards determining which organizational strategies and conditions contribute to better workforce health outcomes, for whom, and under what circumstances.

## Figures and Tables

**Table 1:** Overview of the included literature.

Characteristic	*n*
Included reports	51
Publication period	1999–2025
Geographical distribution	
Iran	15
Taiwan	11
South Africa	3
Pakistan	2
Other countries, multicountry, or international contributions	20
Primary narrative domain	
Implementation conditions	17
Standards and governance	14
Workforce interventions/outcomes	14
Conceptual foundations	6
Study design	
Cross-sectional surveys/organizational self-assessments	26
Qualitative or predominantly qualitative studies	10
Conceptual or narrative review contributions	9
Other designs	6

Notes: HPH = Health Promoting Hospitals; HPH&HS = Health Promoting Hospitals and Health Services. Categories under primary narrative domain reflect the principal contribution assigned to each report for the purposes of the narrative synthesis. Some publications originated from overlapping projects, hospital networks, or study populations; therefore, the 51 reports should not be interpreted as 51 independent organizational experiences.

## Data Availability

No new data were created or analyzed in this study. Data sharing is not applicable to this article.

## Abbreviations

The following abbreviations are used in this manuscript:

HPH	Health Promoting Hospitals
HPH&HS	Health Promoting Hospitals and Health Services
WHO	World Health Organization
ASUFC	Friuli Centrale Healthcare University Trust
MOPR	Mindfulness-Oriented Professional Resilience

## Appendix A

**Table A1:** Characteristics of the included reports.

Primary Narrative Domain/Reference	Country and Setting	Study Design	Population/Sample	HPH Framework/ Exposure	Workforce Focus	Main Workforce- Relevant Contribution
Conceptual foundations [2]	International/Europe—WHO HPH model project, European Pilot Hospital Project, international HPH network	Conceptual/developmental paper	Not applicable (conceptual synthesis of HPH development)	WHO HPH framework; Ottawa Charter; Budapest Declaration; Vienna Recommendations; patient, staff, organization and community domains	Staff explicitly positioned as a core HPH target group; staff health promotion is integrated with organizational development and quality improvement.	Foundational source establishing workforce health as an intrinsic component of the HPH model and linking HPH to organizational development.
Conceptual foundations [9]	International—Hospital nursing/HPH literature	Critical narrative review/discussion paper	Literature-based; no primary sample	WHO HPH movement; Ottawa Charter; Vienna Recommendations	Focuses on hospital nurses as the largest professional workforce and their role in organizational health promotion reform.	Shows the workforce not only as a recipient of health promotion but also as an active agent of organizational HPH transformation.
Implementation conditions [56]	France/Europe—European healthcare settings and HPH policy context	Narrative literature review + policy analysis	Not applicable (literature/policy sources)	HPH concept in France and Europe; organizational strategies and partnerships; European health policy	Workforce is addressed through organizational strategies, partnerships and the need to develop professional health-promotion skills.	Early implementation-oriented source showing that workforce capacity alone is insufficient without coherent organizational and policy support.
Conceptual foundations [57]	International—Hospitals/HPH/salutogenesis	Conceptual chapter with targeted literature review	Not applicable; literature on patients and hospital staff	HPH framework linked to salutogenesis; six intervention areas for staff; organizational-development and quality-improvement principles	Staff are a defined HPH target group; chapter addresses SOC, staff workability, healthy workplace design and staff-oriented salutogenic interventions.	Provides the theoretical bridge between salutogenesis and HPH workforce well-being and specifies organizational resources needed to operationalize it.
Standards and governance [11]	International—HPH literature, 1997–2016	Systematic review structured with EFQM	23 included publications	WHO HPH framework; EFQM-WHO model; HPH implementation and development criteria	Employee Results include healthy workplace, staff satisfaction, skills, participation, support, welfare and healthy lifestyle.	Provides a structured synthesis of organizational enablers and workforce-related results, useful for the implementation/barriers–facilitators domain.
Conceptual foundations [3]	Australia—Major specialist hospital in Adelaide plus international/Australian HPH literature	Mixed conceptual/empirical paper: literature review, observation and action-research case study (1994–1998)	One major specialist Adelaide hospital; additional observational information from Australian hospitals	Settings approach/HPH; four organizational types from isolated project to health-promoting setting + community orientation	Staff health is one of the core activity categories; staff also become organization-wide actors in more mature HPH types.	Foundational typology for distinguishing individual projects from genuine organizational transformation—central to interpreting workforce initiatives in HPH.
Standards and governance [52]	Lithuania—Three randomly selected hospitals in the Lithuanian HPH network (large, medium, small)	Cross-sectional questionnaire survey	1019 health professionals: 280 physicians and 739 nurses; includes managerial staff	Five WHO HPH standards, including Standard 4 promoting a healthy workplace	Direct assessment of health professionals’ awareness and perceived applicability of HPH standards; Standard 4 explicitly concerns staff workplace health.	Empirically demonstrates that the workforce dimension of HPH is partly an implementation-capacity issue: staff knowledge and organizational embedding affect perceived applicability.
Workforce interventions/outcomes [29]	Taiwan—Nationwide survey of 100 hospitals; certified HPH vs. non-HPH hospitals	Nationwide cross-sectional comparative study	70,622 completed questionnaires were received (71.5% response rate); 33,592 respondents were full-time licensed registered/practical nurses. After restricting the cohort to nurses aged 18–65 years working in five main hospital units and excluding incomplete records, 26,011 nurses were analyzed (14,769 in certified HPH hospitals; 11,242 in non-HPH hospitals).	Certified Health Promoting Hospital status/Taiwan HPH implementation	Direct workforce-health study comparing nurses’ health practices by hospital HPH status.	One of the strongest large-scale empirical workforce-outcome studies linking HPH status with staff health behavior.
Conceptual foundations [12]	International/Canada-oriented conceptual discussion—Hospitals as healthy organizations and community health-promoting institutions	Conceptual/perspective article	Not applicable	Ottawa Charter; healthy hospital vs. health-promoting hospital; healthy organization framework	Healthy workplace for staff is a defining element of a ‘healthy hospital’; staff work life is linked to organizational culture, physical environment and organizational capacity.	Early conceptual articulation of the ‘healthy hospital’ as both a healthy workplace and an organizational system supportive of staff health.
Workforce interventions/outcomes [45]	Canada (Quebec)—University hospital implementing HPH; health-promoting workplace subproject for nurses	Exploratory organizational case study using questionnaire, semi-structured interviews and document analysis	Strategic actors in a university hospital; 7 key actors interviewed; focus on nursing workplace practices	WHO HPH project; WHO self-assessment standards adapted to Quebec; health-promoting workplace dimension	Direct focus on nurses and organizational practices compatible with a health-promoting workplace.	Strong organizational case showing that workforce health promotion requires participatory governance and policy integration, not only wellness activities.
Workforce interventions/outcomes [32]	Taiwan—Taiwan HPH Network; literature and national HPH data	Structured narrative review/regional evidence synthesis	Seven eligible published studies (plus gray literature); includes nationwide staff survey data from 100 hospitals	WHO HPH movement; four HPH perspectives: patients, staff, organization, community	Staff health is one of the four explicit HPH perspectives; synthesis includes staff health behaviors, workplace violence, staff participation and staff-oriented services.	Key synthesis linking Taiwan’s HPH organizational development with staff health outcomes and implementation conditions.
Implementation conditions [21]	Iran—10 educational hospitals affiliated with Tabriz University of Medical Sciences	Q-methodology mixed qualitative–quantitative study	33 health professionals: 24 nurses, 7 physicians, 2 managers	WHO HPH initiative and standards; implementation/adoption focus	Frontline health professionals were the source of implementation perspectives; several barriers directly concern workload, commitment, knowledge and workforce capacity.	Strong empirical source for the barriers/facilitators theme, especially biomedical bias, workload, organizational resources and staff commitment.
Workforce interventions/outcomes [47]	Estonia—All Estonian hospitals surveyed; comparison of HPH-network and non-HPH hospitals	Cross-sectional postal survey of hospital top managers	54 hospitals targeted; 20 were HPH members (final response numbers reported in paper)	WHO HPH standards; Estonian HPH Network; comparison with national quality-assurance requirements	Direct healthy-workplace component: workplace risk analysis, staff job-satisfaction studies, staff satisfaction, psychosocial stress, work environment and health risks.	Important comparative evidence that HPH hospitals more often institutionalized occupational risk analysis and staff-satisfaction monitoring.
Workforce interventions/outcomes [14]	Iran—Shahid-Beheshti educational hospital, Maragheh University of Medical Sciences; described as a health-promoting hospital	Best-practice implementation/audit-and-feedback project using JBI PACES and GRiP	86 nurses at baseline audit; 62 nurses at follow-up audit	Hospital identified as an HPH setting; intervention itself based on JBI evidence-based violence-management criteria rather than WHO HPH standards	Direct nurse occupational-health/safety intervention addressing workplace violence, staff injury, knowledge, support and safe environment.	Concrete implementation example linking workforce safety and violence management to an HPH hospital context.
Implementation conditions [44]	Pakistan—Karachi; public and private tertiary hospitals plus health-policy and agency stakeholders	Qualitative study using key-informant interviews and focus groups	11 key informants + 2 focus groups of 6 participants each (3 nurses, 3 doctors per group)	WHO five HPH core components/standards adapted to Pakistan	Staff healthy-workplace needs are explicitly analyzed: safety/security, health screening, flexible work arrangements, recreation and participation in decisions.	Strong qualitative evidence that healthy-workplace implementation in resource-constrained settings depends on basic safety, staff empowerment and policy support.
Standards and governance [26]	Iran—Madani Heart Center, Tabriz, Northwest Iran	Cross-sectional HPH self-assessment survey	258 analyzed staff questionnaires (270 returned; 12 incomplete); 67.4% clinical, 32.6% administrative staff	WHO HPH 40-item self-assessment tool; five standards including 10-item healthy-workplace standard	Staff both assess HPH implementation and are the target of Standard 4 (promoting a healthy workplace/workforce).	Useful standards-based evidence showing moderate implementation but persistent weaknesses in management and workforce-oriented structures.
Workforce interventions/outcomes [39]	Taiwan—100 hospitals nationwide	Nationwide cross-sectional survey	26,979 nurses	Certified HPH status and ‘outstanding HPH’ performance as hospital-level exposure	Direct occupational-safety outcome among nurses: physical and non-physical workplace violence.	Large-scale workforce outcome study showing that the quality/intensity of HPH implementation may matter more than certification status alone.
Workforce interventions/outcomes [28]	Iran—Specialized hospitals in Hamadan	Cross-sectional original research with PLS structural equation modeling	600 nurses: 300 from each of two specialized hospitals in Hamadan	HPH framework; workforce/healthy-workplace dimension used to build a model of personnel health and job satisfaction	Direct workforce well-being model integrating lifestyle, quality of life, organizational culture and job satisfaction.	One of the most directly relevant empirical models connecting personal health resources and organizational culture to workforce job satisfaction within an HPH-oriented framework.
Standards and governance [15]	Iran—9 educational hospitals in Isfahan	Cross-sectional organizational self-assessment	9 hospitals; multidisciplinary assessment teams involving accreditation/quality staff and other hospital professionals	WHO HPH self-assessment tool; five standards including healthy workplace	Staff health promotion is one of five identified activity categories and Standard 4 is explicitly assessed.	Early Iranian standards-based assessment documenting uneven HPH development and the need for stronger organizational integration.
Standards and governance [24]	Iran—Farshchian Heart Center, Hamadan; HPH network member since 2016	Cross-sectional evaluation of WHO HPH standards	111 hospital employees, 109 patients, 6 management staff	International HPH&HS Network member; WHO five HPH standards	Hospital employees and management are explicit respondent groups; healthy-workplace and staff health-promotion awareness/participation are central.	Important implementation-gap study showing the distance between HPH certification/membership and actual staff awareness and participation.
Implementation conditions [40]	South Africa—Rural district hospital, Limpopo Province; pilot transformation into a WHO-Europe HPH	Substantive commentary/implementation case report grounded in critical action research and PRECEDE–PROCEED	Hospital staff, patients, relatives and community stakeholders; detailed staff sample reported in companion project paper U035	WHO-Europe HPH standards; Ottawa Charter; organizational transformation of hospital structure and culture	Staff well-being and a healthy workplace are explicit targets; staff empowerment/training and integration into organizational routines are central.	Early African implementation example demonstrating that workforce well-being was treated as part of whole-hospital transformation rather than as a standalone wellness project.
Implementation conditions [48]	Ghana—Shai-Osudoku District Hospital, Greater Accra Region	Qualitative organizational case study using interviews, open-ended questionnaires and direct observation	Multidisciplinary hospital staff: doctors, administrators, nurses, public-health practitioners, HP focal persons, pharmacists, physician assistants and allied health professionals; exact participant count not clearly reported	WHO HPH model + ADKAR change-management model; workforce well-being explicitly included in HPH framework	Workforce implementation conditions are central: workload, staffing, motivation, teamwork, training, management support, working hours and professional collaboration.	Strong recent evidence for the implementation theme, particularly workload, staffing, motivation, professional silos and management support within an explicit HPH + ADKAR framework.
Implementation conditions [41]	South Africa—252-bed rural district hospital, Limpopo Province; 791 total staff	Organizational case study/action-research implementation project with diagnostic needs assessment	Needs assessment: 295 hospital staff, 145 patients, 63 relatives; hospital total staff 791	WHO-Europe HPH standards and self-assessment tool; organizational diagnosis; HPH integrated into quality assurance	Staff are a direct target of needs assessment, empowerment training, healthy-workplace development and organizational participation.	Detailed companion case demonstrating a full organizational pathway from workforce needs assessment to empowerment, healthy-workplace actions and institutionalization.
Standards and governance [20]	Iran—10 educational hospitals affiliated with Tabriz University of Medical Sciences	Cross-sectional study of HPH adaptability/applicability	354 health professionals: physicians, nurses and general managers	Validated WHO HPH self-assessment tool; five standards including healthy workplace	Health professionals are respondents and Standard 4 directly assesses healthy/safe workplace and staff health-promotion activities.	Provides empirical evidence of limited perceived applicability of HPH standards, including the healthy-workplace dimension; applicability ratings varied according to prior HPH knowledge and hospital characteristics.
Implementation conditions [35]	Taiwan—Taiwan HPH Network; 52 HPH hospitals responding	Cross-sectional organizational-capacity survey informed by the Burke–Litwin model	52 HPH coordinators from 55 eligible hospitals (94.5% response)	HPH standards and organizational capacity building; Burke–Litwin transformational/transactional framework	Staff participation is one of seven explicit organizational-capacity dimensions; staff training, job descriptions, performance systems and support are assessed.	Central organizational-change paper showing why formal HPH adoption may remain additive unless everyday systems and staff participation are transformed.
Workforce interventions/outcomes [33]	Taiwan—All hospitals committed to the Taiwan HPH approach by end-2009	Cross-sectional survey of healthcare workplace health promotion capacity	52 responding hospitals of 55 eligible HPH hospitals	Taiwan HPH initiative; workplace health promotion as staff-focused HPH domain	Direct focus on workplace health promotion, staff participation and staff-oriented health services.	Direct evidence that Taiwan’s HPH movement expanded workplace-health infrastructure and staff participation at hospital level.
Implementation conditions [46]	China—Beijing; 106 hospitals, including HPH pilot and control hospitals	Comparative cross-sectional evaluation using questionnaires and interviews with managerial staff	281 managerial employees: 93 from pilot HPH hospitals and 188 from control hospitals	Beijing HPH pilot based on Ottawa Charter and WHO HPH development principles	Managerial workforce knowledge, professional skills and allocation of staff/personnel are treated as central implementation resources.	Important comparative evidence positioning leadership and workforce resources as prerequisites for HPH institutionalization.
Implementation conditions [34]	Taiwan—Taiwan HPH Network; hospitals committed to HPH by end-2009	Cross-sectional questionnaire study of implementation barriers/facilitators and organizational capacity	52 HPH hospitals of 55 eligible (HPH coordinators as respondents)	HPH implementation; organizational capacity building; leadership, systems and staff-participation dimensions	Staff participation is a major analytic domain; detachment, professional interest, incentives, knowledge/skills and duty conflict are directly examined.	One of the strongest empirical sources for recurrent barriers/facilitators, particularly staff detachment, leadership, resources, incentives and institutional integration.
Implementation conditions [37]	Taiwan—Dalin Tzu Chi Hospital; single HPH organizational case	Qualitative organizational case study with triangulation and thematic analysis	Single hospital; multiple organizational data sources and key informants during a 3-month field period plus continued follow-up	WHO HPH initiative; organizational capacity building; Burke–Litwin transformational/transactional concepts; addition vs. integration models	Staff participation, workload, incentives, effort–reward balance, professional engagement, training and staff-oriented HP practices are central.	Rich case evidence explaining how organizational capacity, incentives and workforce participation interact during HPH implementation.
Standards and governance [51]	Japan—Japan Association for Development of Community Medicine: 25 hospitals, 48 clinics and 18 nursing-care facilities	Organization-wide web survey with telephone/email follow-up; cross-sectional implementation assessment	91 facilities; 100% response	Activities categorized against the 2020 HPH Standards; patient/user, community and staff target domains	Staff-directed health-promotion activities are explicitly measured across facility types; workload is analyzed as an implementation barrier.	Recent 2020-standards evidence confirming workload as a recurrent barrier and organizational infrastructure as a key facilitator for staff-focused health promotion.
Implementation conditions [38]	Taiwan—National survey of hospitals above local community-hospital level	National cross-sectional organizational survey	162 valid hospital responses from 474 hospitals (34.18%)	HPH/health-promoting organization framework; Organizational Health of Hospital Assessment Scale (policy/leadership, physical environment, healthy culture, health resources/activities)	Employees’ and organizational health are explicit conceptual targets; staff HP training is a measured organizational mechanism.	Strong national evidence linking whole-organization HPH capacity with organizational effectiveness and identifying staff training as an implementation mediator.
Workforce interventions/outcomes [13]	Czech Republic—General University Hospital in Prague; one of the country’s largest hospitals and HPH network member	Repeated cross-sectional staff survey (2012 vs. 2023)	All hospital employees invited; response rates 25% (2012) and 28% (2023); physicians, nurses and other personnel	International HPH project/network membership; smoke-free hospital and tobacco-control activities implemented within HPH	Direct workforce-health monitoring of tobacco/nicotine use among physicians, nurses and other staff.	Rare long-term workforce behavioral outcome study within an explicit HPH organizational tobacco-control program.
Workforce interventions/outcomes [55]	Multicountry—36 clinical hospital departments in HPH member hospitals across eight countries/regions	Randomized controlled implementation trial at department level	36 departments analyzed; 5285 patients; 2529 staff members; 1750 medical records	WHO HPH standards + recognition process + Fast-IM; PDCA and structured strategic implementation	Staff health status was a prespecified outcome; staff were also part of the intervention setting and data collection.	Methodologically strongest implementation study in the corpus; separates implementation success from actual staff-health effects.
Standards and governance [18]	Iran—Two specialized hospitals affiliated with Hamadan University of Medical Sciences	Applied cross-sectional HPH self-assessment	Two hospitals; assessment based on WHO HPH checklist and hospital evidence	WHO HPH five standards, including promoting a healthy workplace	Healthy-workplace standard directly assesses staff-oriented organizational conditions.	Clear standards-based evidence that workforce-oriented HPH components can lag behind patient-facing dimensions.
Implementation conditions [49]	Greece—Aretaieion University Hospital, Athens; WHO European Pilot HPH Project (1993–1997)	Program evaluation using professional and patient questionnaire surveys	44 professionals invited, 39 responded: 24 doctors, 12 nurses, 1 psychologist, 2 medical students; 84 patient respondents	WHO European Pilot Project of Health Promoting Hospitals; Vienna Recommendations	Staff were respondents and one explicit HPH target; workforce-related outcomes included training, teamwork and working conditions.	Foundational empirical pilot evidence linking HPH implementation with staff training, teamwork and perceived improvements in working conditions.
Implementation conditions [36]	Taiwan—55 hospitals committed to HPH; 52 responding hospitals	Cross-sectional retrospective self-report of perceived pre/post organizational change	52 HPH coordinators (one per responding hospital)	HPH initiative as organizational-capacity-building strategy	Staff participation and individual knowledge/skills are explicit strategy domains; staff-targeted changes are included within the HPH stakeholder model.	Documents coordinators’ perceived organizational-capacity changes following HPH adoption, while showing that reported changes were more prevalent for patient and community domains than for staff.
Implementation conditions [43]	Pakistan—Karachi healthcare system; public/private hospitals, policy makers and UN/donor stakeholders	Qualitative exploratory study using key-informant interviews and focus groups	Purposively selected hospital administrators, healthcare providers, policy makers and UN/donor representatives; exact *n* to verify from full text if needed	WHO HPH concept and settings approach	Participants explicitly perceived benefits for hospital staff, including a healthier/happier workplace and workforce retention; training needs for providers were emphasized.	Provides pre-implementation stakeholder evidence that staff well-being and retention are perceived organizational benefits of HPH in a developing-country context.
Implementation conditions [19]	Iran—Two hospitals in Hamadan; medical staff from multiple professions	Qualitative content-analysis study using framework analysis	55 analyzed interviews with nurses, midwives, physicians and paramedics	Interview guide explicitly based on WHO HPH standards; healthy-environment standard prioritized because staff were its target group	Entire study is workforce-centered: healthy workplace, staff empowerment, HPH culture, staff performance and participation.	One of the most directly relevant qualitative studies in the corpus, integrating healthy workplace, staff skills, culture, participation and unintended workload effects.
Standards and governance [50]	Indonesia—Private Class C hospital in Depok City; hospital HPH unit	Descriptive cross-sectional self-assessment study	20 HPH-unit health workers: 12 nurses, 4 laboratory staff, 2 midwives, 1 physiotherapist, 1 pharmacist	2018 Indonesian Ministry of Health HPH standards adapted from WHO HPH; four standards: policy/management, assessment, intervention, monitoring/evaluation; discussed against WHO 2006/2020 standards	Standards explicitly cover patients, families, staff, visitors and community; paper notes minimal attention to healthcare workers in practice and recommends staff training.	Recent standards-based evidence illustrating the implementation gap between formal inclusion of staff and actual workforce-focused health promotion.
Workforce interventions/outcomes [30]	Taiwan—100 hospitals nationwide	National cross-sectional survey	21,095 full-time nurses	Health-promoting-hospital certification included as a hospital-level covariate in nationwide staff-health analysis	Direct workforce-health study of nurses across hospital units; assesses physical health, depressed mood and occupational hazards.	Very large national workforce-health study defining the heterogeneity of staff needs that HPH workplace programs should address.
Implementation conditions [58]	Belgium (French-speaking community)—French-speaking Belgian hospitals/healthcare organizations; survey status in 1998 with reference to the WHO-Europe HPH network	Cross-sectional institutional survey/implementation status review	Hospitals and healthcare organizations in the French Community of Belgium; exact response denominator not clearly recoverable from the article text	WHO-Europe Health Promoting Hospitals project; Budapest Declaration; Ottawa Charter	HPH objectives explicitly include healthy working conditions for staff, staff health education, staff needs assessment and staff participation in institutional discussion forums.	Historical implementation source showing that time/resources, professional motivation and formal organizational support were already recurrent determinants of HPH development.
Implementation conditions [54]	Multicountry—Clinical departments in four countries participating in the WHO-HPH Recognition Process RCT	Nested qualitative study using semi-structured key-informant interviews and inductive content analysis	45 key informants: 14 managers, 14 physicians, 13 nurses, 4 other clinical staff	WHO-HPH Recognition Process; WHO standards/indicators; strategic implementation process nested in RCT	Staff and managers are the primary informants; study focuses on their implementation experiences, learning and organizational/cultural change.	Qualitative companion to the RCT explaining how staff experienced organizational HPH implementation and why process design influences uptake.
Standards and governance [53]	Germany (with holding sites also in Austria)—Three acute-care hospitals in four locations within the Immanuel Diakonie Group; >2000 staff across holding	Five-year multi-method strategy-development and implementation project	CEO, 20 senior doctors/managers, working-group leaders and hospital staff; organization-wide staff value/satisfaction surveys	WHO HPH Standards and 18 HPH strategies integrated with EFQM and Balanced Scorecard	Staff are explicit stakeholders; organization-wide staff satisfaction/value surveys and staff involvement are integrated into strategy monitoring and implementation.	Key governance paper demonstrating how HPH principles can be translated from declarations into measurable organizational strategy using BSC/EFQM.
Standards and governance [17]	Iran—Three selected military hospitals in Tehran	Descriptive-analytical cross-sectional survey	400 nursing staff selected by quota and simple random sampling	WHO HPH questionnaire across five standards, including healthy workplace	Nurses are the respondent population and healthy workplace is an explicit HPH standard.	Large nurse-based readiness study showing that workforce-oriented Standard 4 remains a priority in military hospital settings.
Standards and governance [25]	Iran—All 20 hospitals in Hamadan province; 18 public, 2 private; one international HPH network member	Cross-sectional organizational self-assessment study	20 hospitals	WHO HPH self-assessment questionnaire; five standards including Standard 4 healthy workplace	Healthy workplace directly assesses staff health/safety, policy awareness, professional development, participation, smoking cessation and annual staff surveys.	Strong contemporary standards-based evidence that workforce-oriented HPH components lag behind patient-facing domains.
Standards and governance [23]	Iran—Four selected hospitals	Mixed-method case study: quantitative importance–performance analysis followed by focus groups	Hospital managers/officials participating in WHO HPH assessment across four hospitals; exact total *n* not clearly stated in retrieved text	WHO HPH 40-item questionnaire; five standards; importance–performance analysis	Healthy workplace is a central standard, and qualitative improvement strategies specifically target staff education, lifestyle and participation.	Useful bridge between HPH self-assessment and concrete workforce-oriented improvement planning.
Workforce interventions/outcomes [31]	Taiwan—100 hospitals nationwide	National cross-sectional staff survey	59,396 full-time hospital employees: 4202 physicians, 31,639 nurses, 2315 pharmacists, 8161 other health professionals, 13,079 administrative staff	Taiwan HPH Network/WHO HPH staff-health emphasis; hospital-based workplace health promotion activities	Direct workforce-health study comparing multiple occupational groups and participation in hospital HPH activities.	Largest multi-professional workforce study in the corpus, demonstrating occupational inequalities in both health behaviors and participation.
Standards and governance [16]	Iran—Nine educational hospitals/medical education centers in Isfahan	Descriptive cross-sectional HPH self-assessment	9 educational hospitals	WHO HPH self-assessment standards	Workforce-related elements include staff awareness of HPH policy, induction training, smoking cessation and healthy-workplace implementation.	Follow-up Isfahan evidence emphasizing persistent staff-awareness, training and smoking-cessation gaps in HPH implementation.
Conceptual foundations [22]	Iran—38 selected hospitals across five provinces	Descriptive cross-sectional instrument-development study using EFA and CFA	280 participants: 100 nurses, 129 managers, 51 academic faculty members	WHO HPH conceptual framework organized into patient, staff, organization and community health-promotion dimensions	Staff Health Promotion is one of four explicit latent dimensions, including lifestyle empowerment, self-care, participation and safety/health education.	Important measurement/model paper empirically confirming Staff Health Promotion as a distinct HPH domain in an Iranian hospital sample.
Workforce interventions/outcomes [42]	South Africa—Rural district hospital transformed into an HPH; Limpopo Province	Conference paper reporting cross-sectional needs assessment within participatory action-research HPH intervention	295 hospital staff, 212 patients and 63 relatives	Health Promoting Hospital settings approach; PRECEDE–PROCEED; same rural HPH transformation project as U033/U035	Direct measurement of staff health behaviors and health-education/promotion needs.	Provides the most direct quantitative staff-health data from the South African HPH pilot and links needs assessment to intervention design.
Workforce interventions/outcomes [27]	Iran—Tabriz Shahid Madani Heart Center; beginning of HPH-standard implementation	Descriptive-analytical cross-sectional staff survey	319 administrative and therapeutic hospital employees	Study explicitly conducted at the beginning of activities to implement HPH standards	Direct workforce-health behavior assessment, especially among nurses and other hospital employees.	Direct baseline workforce-health study linking HPH implementation to concrete lifestyle and stress-management needs among hospital employees.

Note. HPH = Health Promoting Hospitals; HPH&HS = Health Promoting Hospitals and Health Services; WHP = Workplace Health Promotion. Reports arising from overlapping projects, hospital networks, or study populations were retained when they contributed complementary information and should not be interpreted as independent organizational experiences.
